# Intraoperative Fascial Traction - From Concept to Comprehensive Application

**DOI:** 10.3389/jaws.2026.16018

**Published:** 2026-02-06

**Authors:** H. Niebuhr, G. Woeste, C. Winkler, S. Behle, W. Reinpold, H. Dag, F. Köckerling

**Affiliations:** 1 Hamburg Hernia Center, Hamburg, Germany; 2 Agaplesion Elisabethenstift Darmstadt, Darmstadt, Germany; 3 Fasciotens GmbH, Essen, Germany; 4 Hernienzentrum Vivantes Humboldt-Klinikum Berlin, Berlin, Germany

**Keywords:** abdominal wall reconstruction, fascial traction, fasciotens, IFT, ventral hernia repair

## Abstract

Intraoperative Fascial Traction (IFT) represents a promising alternative technique for complex abdominal wall reconstruction in large ventral hernias, particularly those exceeding 10 cm in width. Developed by Swiss and German surgeons and introduced clinically in 2021, IFT achieves fascial closure without extensive muscle component separation. Multiple studies demonstrate closure rates of 79%–96% for defects below 19 cm, though rates decline significantly for larger defects. Preoperative botulinum toxin A (BTA) administration and transversus abdominis muscle release (TAR) are often combined with IFT. The paper discusses the Hamburg algorithm 2.0 as it provides a structured treatment approach based on defect width, recommending IFT as a first-line intervention for defects up to 15 cm and incorporating additional component separation for larger hernias. Controlled fascial traction allows standardised treatment and can lead to higher fascial closure and lower recurrence rates.

## Introduction

It is generally accepted that two key principles guide modern ventral hernia repair: 1. The hernia repair should be augmented by a mesh in retrorectus, retromuscular or preperitoneal position and 2. The fascial defect should be closed primarily by suture and bridging should be avoided [1].

Around the millennium, the Dutch working group of Hans Jeekel from Rotterdam showed that a mesh augmented incisional hernia repair is superior to direct suture repair in terms of recurrence rates [2, 3]. Later, other Randomised Controlled Trials demonstrated that a mesh in sublay position has significantly lower recurrence and seroma rates as mesh augmentation in Onlay position [4, 5]. Regarding fascial closure, it was shown that a bridging of the anterior rectus sheath (ARS) has drastically increased risk of recurrence [6, 7].

Naturally, it is relatively easy to follow these key principles in smaller hernias. The more challenging cases are summarised as complex abdominal wall hernias. Although the term is not standardised, it often involves a hernia width above 10 cm or significant loss of domain [8]. Those cases require extensive reconstruction of the abdominal wall, referred to as complex abdominal wall repair (CAWR). For this purpose, component separation techniques such as anterior component separation (ACS) and transversus abdominis muscle release (TAR) have been available for many years and have proven to be very valuable [1, 9–11]. Nevertheless, apart from being surgically demanding, they have in common that the aponeurosis or muscle fibres of one or more of the lateral abdominal wall muscles are deliberately severed. Looking for alternatives to restore the abdominal wall and to achieve fascial closure without extensive preparation, Eucker et al from Switzerland started using fascial traction for CAWR in 2012 and published their first case series in 2017 [12]. At the same time the conceptualization of vertical fascial traction using an external device was developed by Lill from Germany, first proven in an animal model and afterwards used for open abdomen treatment [13, 14]. After fusing the concepts, intraoperative fascial traction (IFT) was introduced as a new technique in CAWR and the first case series was published in 2021 by Niebuhr et al. showing promising results [15].

## Literature Review

Since the introduction of the concept by Eucker et al. in 2017 several publications have shown the outcomes of IFT. The first paper on abdominal wall expander system (AWEX) including 10 patients with a median hernia width of 12.0 cm reported a closure rate of 60% and a mean reduction of fascia-to-fascia distance of 8.5 cm [12]. In 2022 follow-up data on AWEX were published including 33 cases. Median hernia width in this cohort was 13.0 cm. Complete fascial closure was achieved in 20 cases (60.6%) and 1 recurrence (3%) after a median follow-up of 29 months was reported [16]. Also showing promising data, performing AWEX using a non-standardised, “self-built” mechanism seems to lead to a relatively low closure rate. In 2021, first results were published for large incisional hernia repair using fasciotens^®^Abdomen (Fasciotens GmbH, Germany) which was initially developed for open abdomen treatment allowing to apply controlled, reproducible and quantifiable traction. A total of 21 patients with a mean intraoperative measured defect width of 17.3 cm were included in this prospective observational trial. 13 patients were pretreated with Botulinum Toxin A (BTA). An overall closure rate of 100% and Surgical Site Occurrence (SSO) rate of 19% was reported [15]. The results were confirmed in a retrospective analysis of 50 cases showing a closure rate of 90% and SSO rate of 12% [17]. Most recently, Woeste et al. published the results of a follow-up of 100 patients treated with IFT [18]. The mean follow-up time was 19.6 months in this cohort. BTA was administered preoperatively in 87% of the cases and TAR was added in 28%. On average, the defect size was 15.8 cm and fascial closure was achieved in 94% of all cases. The SSO rate was 33%, however 54.5% were seromas. Having a relatively small mean mesh width of 22.6 cm, a recurrence rate of only 2% was reported, which may emphasise the importance of ARS closure.

The clinical effect of IFT in terms of medialisation of the lateral abdominal wall was also confirmed in a cadaver study [19]. Retrorectus dissection was first carried out in a total of four fresh frozen specimens. Subsequently, IFT was applied for 30 min. The mean medial advancement was 10.5 cm, which is in line with clinical findings [15, 17, 20]. In 2024, we reported on 143 cases treated at the Hamburg Hernia Centre [20]. All patients in this cohort received Botulinum Toxin A 4 weeks prior to surgery and IFT was also used in all cases. The mean hernia width was 16.9 cm and 68.5% of the patients had a transverse hernia size above 15 cm. The patient cohort was divided into subgroups as shown in Table 1. It shows that closure rates of 95% can be achieved for cases up to 15 cm of hernia width. However, closure rates decline especially above 19 cm and bridging becomes more likely even with higher TAR rates (48.6% in subgroup 3).

**TABLE 1:** Subgroup analysis in relation to hernia defect width based on Niebuhr et al., Springer Hernia [20]. *SSO* surgical site occurrences, *SSI* surgical site infections, *TAR* transversus abdominis muscle release.

​	Subgroup 1	Subgroup 2	Subgroup 3
Number of patients (total number = 143)	45	61	37
Defect width [cm]	8 - >15	15–19	>19
Lateral or additional lateral defect rate	4/45 (8.9%)	7/61 (11.5%)	4/37 (10.8%)
Closure rate	43/45 (95.6%	48/61 (78.7%)	12/37 (32.4%)
TAR rate	7/45 (15.6%)	18/61 (29.5%)	18/37 (48.6%)
Intraoperative complication rate	0/45 (0%)	2/61 (3.3%)	3/37 (8.1%)
SSO	11/45 (24.4%)	17/61 (27.9%)	15/37 (40.5%)
SSI	4/45 (8.9%)	6/61 (9.8%)	9/37 (24.3%)
Re-operation	5/45 (11.1%)	10/61 (16.4%)	7/37 (18.9%)

## Hamburg Algorithm 2.0

**FIGURE 1:**
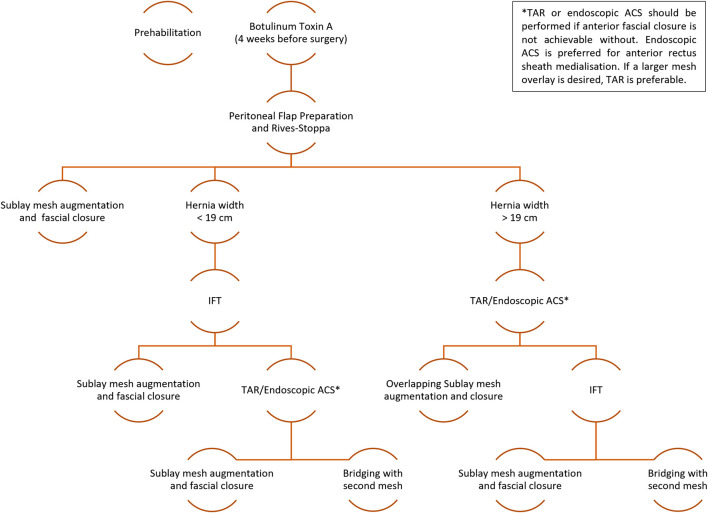
The Hamburg algorithm 2.0 for complex abdominal wall repair. IFT, Intraoperative Fascial Traction; TAR, Transversus Abdominis Muscle Release; ACS, Anterior Component Separation.

Based on the results an initial Hamburg algorithm for CAWR was presented. Since then, we have further adapted the algorithm following our ongoing clinical experience and feedback from conferences and discussions with colleagues. We now propose an approach based on the subgroups for the treatment of ventral hernias in order to address the raising complexity associated with an increase in defect width (see Figure 1). It is important to note that the defect width is only one criterion for the complexity of a hernia and that each patient requires a tailored approach. The decision-making process depends on whether ARS closure can be achieved after each step of the algorithm. The elements of the algorithm will be discussed in detail below.

### Subgroup 1/2 – Hernia Width Below 19 cm

The analysis of 143 patients treated with BTA and IFT has shown that anterior sheath closure rates of 96% for defects with a transverse diameter below 15 cm and 79% for defects below 19 cm in width can be achieved [20]. The procedure for these patients therefore initially consists of peritoneal flap preparation and retromuscular dissection according to Rives-Stoppa [21]. If the anterior layer of the rectus sheath can be closed under moderate tension, IFT is not performed. If fascial closure is not possible, IFT will be carried out. Only if closure is still not possible afterwards component separation is added. In rare cases peritoneal flap closure or mesh bridging should be considered [22].

### Subgroup 3 - Hernia Width Above 19 cm

The patient cohort having a defect width above 19 cm showed a relatively low complete closure rate of around 32% [20]. Therefore, TAR or ACS is performed in these patients before IFT is applied. If closure is still not possible afterwards, peritoneal flap closure or mesh bridging should be considered [22].

## Discussion

### Indication

In our own experience, IFT achieves a different range in terms of medialisation of the lateral abdominal wall in a (naturally) heterogeneous patient population. The EHS classification for ventral hernias has strongly helped to standardise hernia characteristics, but every CAWR surgeon knows that each complex hernia is different and needs an individualised treatment plan [1, 23]. Therefore, although it can generally be said that IFT is used in the vast majority of cases for ventral hernias with a diameter above 10 cm, it is difficult to predict the definitive length gain preoperatively. Nevertheless, its use does not have to be determined in advance of the operation; rather, it should be used on demand, namely, at the moment when low-tension closure of the ARS after Rives-Stoppa preparation appears difficult. Additionally, IFT should be considered in any case of CAWR with visceroabdominal disproportion which is often the case in Loss of Domain (LOD) hernias. Hence, it has also been previously used in flank and massive scrotal hernias.

### Prehabilitation

Thorough preoperative assessment and planning are key to reducing postoperative risk for complications and recurrence. In addition to the clinical examination, a CT or MRI scan of the abdomen should be performed at rest and during the Valsalva manoeuvre in order to assess the extent of the hernia and plan the operation. Several risk factors have been identified to negatively influence postoperative outcomes. It is very important to perform an individual risk assessment with each patient and to identify potential improvement. To visualise the risk, the CeDAR (Carolinas Equation for Determining Associated Risks) app, which is available for free in the Apple and Android App Store, can be used. Additionally, the colleagues from the York Abdominal Wall Unit have developed a range of leaflets and easy to understand guidance documents for patients to help prepare for the surgery (available for free on the website: https://www.yorkhospitals.nhs.uk/our-services/organdonation/a-z-of-services/abdominal-wall-reconstruction/).

Prehabilitation covers a wide range of factors and influencing variables, with BMI, smoking and diabetes among the most influential. A BMI below 30 kg/m^2^ should be aimed for as it significantly decreases the risk for SSO and recurrence [24, 25]. However, each patient needs a realistic and individual goal for weight loss in order to achieve a lasting effect in the best-case scenario. The same applies to nicotine cessation. Smoking has a significant impact on SSO, wound infections and recurrence rates and therefore patients should stop at least 4 weeks prior to surgery [26, 27]. As is widely known, diabetes has a strong influence on wound morbidities and infections [28]. As CAWR is associated with extensive preparation and large wound areas, strict preoperative control of diabetes is recommended.

### Botulinum Toxin A

Administration of Botulinum Toxin A in the lateral abdominal wall muscles to achieve temporary muscle relaxation was first described in 2009 [29]. Different dosages and number of administration points have been described [29–31]. Hernia defect width reduction was reported to be between approx. 4–6 cm [31]. In our practice BTA is mainly used in cases above 10 cm in width. However, it should be noted that BTA administration in the abdominal wall is an off-label use. The FDA issued a mandated black box warning already back in 2009 concerning the risk of botulism-like symptoms such as muscle weakness, dysphagia and aspiration which can be life-threatening [32–34]. Therefore, the use of BTA should be weighed carefully and especially patients with lung disease should be treated very cautiously with BTA [35].

### Peritoneal Flap and Rives-Stoppa

We strongly believe that the retrorectus dissection according to Rives and Stoppa is the foundation of every (midline) CAWR [21]. The technique is elementary and should be understood by every AWR surgeon. Therefore, we will not go into detail about the procedure itself and refer to detailed descriptions elsewhere. [36, 37]. If possible, the hernia sac should always be preserved until complete fascial closure of both rectus sheaths is achieved. We usually perform the preparation of the hernia sac according to the peritoneal flap technique [22]. Contrary to the initial description of the technique, in our approach, we leave 2/3 of the hernia sac on the PRS on one side and 1/3 on the ARS on the contralateral side. Performing IFT usually requires narrower bridging on the ARS if complete fascial closure is not possible. In the majority of cases a complete ARS closure is achievable. In these cases, the hernia sac attached to the ARS is resected.

### Intraoperative Fascial Traction

Applying IFT does not only involve fascial traction but also a certain approach to restoring the abdominal wall. Part of the flap is regularly used to “bridge” the posterior rectus sheath (PSR) if low-tension direct closure is not possible. Early biomechanical research has shown that the PSR is less resistant to pressure forces and more prone to bursting [38]. Therefore, we see it as less important for abdominal wall strength and the main goal should be a reliable layer between the abdominal organs and the augmenting mesh in sublay position. Before applying IFT, the mesh can already be placed. The fixation of the mesh is an ongoing debate that should be done according to house standards. In our practice the mesh is normally only fixated cranially and caudally with one stitch.

After mesh placement, the prosthetic and the (preperitoneal) landing zone are covered with several (2–4) moist abdominal cloths. The traction forces which are applied by an external device (fasciotens^®^Hernia, Fasciotens GmbH, Germany) are distributed on the fascia by using polyfilamentel USP 2 sutures. 6 sutures per side are anchored in the ARS in a U-shape manner. After placing the sutures, they are crossed to mimic a diagonal directed traction. The sutures are connected to the suture holder of the device and fascial traction is carried out. Normally traction forces of approx. 14 kg are applied which can gradually be increased to about 18 kg. IFT should be maintained for about 30 min and traction sutures should be individually retightened every 2 minutes. Otherwise, traction forces cannot be distributed sufficiently on the fascia. It is very important to have complete muscle relaxation during IFT. The muscle tone works as an antagonist and can lead to a poor outcome. After IFT, sutures are disconnected from the external device and uncrossed. The abdominal cloths are removed and ARS closure is carried out according to the concept of small steps–small bites [39–41].

Eucker et al. have shown that promising results can also be achieved using the AWEX system based on a self-built mechanism. However, using an external device which allows quantifiable traction seems to lead to higher closure rates as shown in the literature review section. It should also be considered that quantifiable and reproducible traction helps to standardise treatment and to compare outcomes. It can also reassure patients and might prevent medicolegal consequences in case of complications or misfunction. Following that, even when self-built, surgeons should follow standardisation and at least measure the applied traction forces.

### Component Separation

Any type of CS might be added according to the patient’s needs as outlined above. It should be considered especially in the case of TAR for what purpose it is performed (mesh overlap, additional lateral defect, closure of the PRS). Studies have shown that TAR is less effective in terms of ARS medialisation compared to ACS [42, 43]. Therefore, ACS still plays a role when it comes to anterior fascial closure. To reduce the risk of SSO, a minimally invasive approach has proven useful [44, 45].

### Sublay Mesh Augmentation

There is an ongoing debate regarding mesh size and overlap in CAWR. Some still recommend a 5 cm overlap in all directions although the data showing an advantage were derived from laparoscopic IPOM procedures without fascial closure [46]. A recent report from the Danish national patients registry showed that a 10–15 cm mesh width in open ventral hernia repair seems to have favourable outcomes in terms of long-term recurrence rates [47]. They also mention that “overlap” is not an appropriate term if the hernia gap is closed and the midline is restored (as it was done in all cases of the aforementioned study). Hence, if the fascial defect is closed, a smaller mesh covering only the retrorectus space might be sufficient. Supporting this, the follow-up published by Woeste et al. regarding CAWR using IFT showed a relatively low recurrence rate of only 2% in 100 patients. Interestingly, the mean mesh width in this cohort was 22.6 cm and a recurrence was found in one patient without TAR and in one patient treated with an additional TAR [18].

### Anterior Rectus Sheath Closure

Although IFT can facilitate fascial closure and has shown closure rates above 90% even in large defects, it is noteworthy that the decision to restore the midline or to pursue a direct fascial closure should be taken with caution. In our practice, we have seen cases of elevated intra-abdominal pressure (IAP) directly after surgery but never faced a subsequent abdominal compartment syndrome. Therefore, some colleagues perform intraoperative measurement of IAP [48]. In order to streamline intraoperative decision-making, close communication with the anaesthetists is necessary regarding peak inspiratory pressure (PIP), particularly while performing IFT. Colleagues from Portugal have shown, that an increased PIP after fascial closure can lead to a postoperative abdominal compartment syndrome [49]. Following their algorithm, we have found PIP variations between 1 and 3 mmHg tolerable, variations above 4 mmHg should be closely monitored (admission to ICU postoperatively). The paper states that only PIP variations above 10 mmHg should lead to bridging. However, we recommend being more cautious and might performing bridging even at lower values. The data so far only present small numbers. Therefore, no general recommendations can be made, and the proposed ranges should only be used as a decision-making aid. If bridging is unavoidable, we normally use a second synthetic mesh as an inlay bridging (ARS). To avoid exposure of the mesh in case of subcutaneous infection or seroma, it is helpful to cover the mesh with residual hernia sac in the style of the peritoneal flap technique [22].

### Limitations

The algorithm presented here is primarily based on single-centre data from a specialised hernia centre. In order to create a simple guideline, hernia width was used as the primary decision criterion. Of course, other factors such as BMI, loss of domain, scarring processes and muscular compliance also play an important role and can influence the surgical procedure. Although initial long-term data is available, further studies with longer follow-up are necessary. In addition, there is currently a lack of prospective comparative studies to identify the advantages and disadvantages of IFT in comparison to other CAWR techniques.

### Conclusion

IFT is a promising technique in CAWR to facilitate fascial closure and restoration of the abdominal wall without extensive preparation as with component separation techniques. Naturally, there are limits and restrictions on its use and every patient with a complex hernia needs a tailored approach and should be treated individually. The achievable medialisation of the abdominal wall differs among patients and is influenced by the anatomy and condition of the lateral abdominal wall. Therefore, IFT is one tool in the CAWR surgeon’s toolbox and should only be used by experienced and dedicated AWR units.
